# Early Experience With Artificial Intelligence Software to Detect Intracranial Occlusive Stroke in Trauma Patients

**DOI:** 10.7759/cureus.57084

**Published:** 2024-03-27

**Authors:** Manisha Koneru, Hamza A Shaikh, Daniel A Tonetti, James E Siegler, Jane Khalife, Ajith J Thomas, Tudor G Jovin, Corey M Mossop

**Affiliations:** 1 Department of Neurointerventional Surgery, Cooper Medical School of Rowan University, Camden, USA; 2 Department of Neurointerventional Surgery, Cooper University Health Care, Camden, USA; 3 Department of Neurosurgery, Cooper University Health Care, Camden, USA; 4 Department of Neurology, Cooper University Health Care, Camden, USA; 5 Department of Neurology, University of Chicago Medicine, Chicago, USA

**Keywords:** imaging, computed tomography angiography, ischemic stroke, trauma, stroke, artificial intelligence

## Abstract

Objective

Identifying ischemic stroke is a diagnostic challenge in the trauma subpopulation. We describe our early experience with artificial intelligence-assisted image analysis software for automatically identifying acute ischemic stroke in trauma patients.

Methods

Patients were retrospectively screened for (i) admission to the trauma service at a level one trauma center between 2020 and 2022, (ii) radiologist-confirmed intracranial occlusion, (iii) occlusion identified on computed tomography angiography performed within 24 hours of admission, (iv) no intracranial hemorrhage, and (v) contemporaneous analysis with the large vessel occlusion (LVO) detection program. Baseline characteristics, stroke detection, response-activation, and outcome data were summarized.

Results

Of 9893 trauma patients admitted, 88 (0.89%) patients had a cerebral stroke diagnosis, of which 10 patients (10/88; 11.4%) met inclusion criteria. Most patients were admitted following a fall (8/10; 80%). Six (6/10; 60.0%) patients had LVOs. The program correctly detected 83.3% (5/6) of patients, and these patients were triaged in less than one hour from arrival on average. The program did not falsely identify non-LVOs as LVOs for any patients.

Conclusions

Identifying adjunct tools to aid timely identification and treatment of ischemic stroke in trauma patients is necessary to increase the chances for meaningful neurological recovery. Our early experience exhibited potential for using automated software to aid occlusion identification and subsequent stroke team mobilization. Future studies in larger cohorts will expand upon these preliminary findings to establish the accuracy and clinical benefit of automated stroke detection tool integration for the trauma population.

## Introduction

Ischemic cerebrovascular stroke in the setting of acute trauma has a significant impact on neurological recovery and overall quality of life. Trauma-related stroke has been reported to occur in 0.004% - 2.5% of trauma patients, with approximately 37% detected on the day of trauma [1,2]. Although primary head/neck injuries and blunt cerebrovascular injuries are associated with a higher likelihood of post-traumatic ischemic stroke, a pattern of primary stroke events causing consequential traumatic injuries is being increasingly recognized [1,3-5]. However, identifying ischemic stroke is uniquely challenging in the trauma subpopulation. Often acutely decompensating, trauma patients presenting obtunded or sedated (i.e., secondary to intubation) may not exhibit the typical neurological deficits that would prompt further diagnostic stroke workup. This causes substantial delays in the recognition and treatment of acute ischemic stroke in trauma patients [4,5]. 

The treatment paradigm for acute large vessel occlusion (LVO) ischemic stroke is rooted in the “time-is-brain” principle; quicker reperfusion halts additional neuron death and facilitates salvage of a greater volume of brain tissue [6-10]. Current guidelines endorse IV alteplase/tenecteplase thrombolysis and/or mechanical thrombectomy for qualifying LVO patients [11]. However, the approach to stroke treatment in the trauma cohort is nuanced due to concomitant factors. For example, IV alteplase, a common thrombolytic agent with therapeutic benefit if administered acutely in ischemic stroke patients, is generally contraindicated in patients with severe head trauma [12]. Mechanical thrombectomy has not been well-studied in the trauma subpopulation [13]. However, recent case reports and a case series suggest that thrombectomy is feasible and associated with favorable neurological recovery in the setting of severe traumatic injury [5,14]. Given the time-sensitive nature of the treatment, developing adjunct tools to facilitate early identification of LVOs in the diagnostically challenging trauma subpopulation is necessary to increase the chance for meaningful neurological recovery. 

The Viz LVO (Viz.ai, San Francisco, CA, USA) is a commercially available, artificial intelligence-assisted software that is FDA-approved for LVO detection on computed tomography angiography (CTA) [15-17]. The software automatically evaluates CTA images; if a suspected LVO is present, the software notifies neurovascular providers for prioritized imaging review and expedited evaluation [18]. Prior studies have demonstrated quicker arrival-to-treatment times after implementing automated LVO detection software as a result of earlier stroke detection and stroke team mobilization [9,16,19,20]. In the context of the unique diagnostic challenges associated with the trauma subpopulation, automated tools have the potential to augment rapid stroke detection and subsequent treatment delivery. We describe our early experience with Viz LVO for automatically identifying acute ischemic stroke in the trauma population at a level one trauma center.

## Materials and methods

Data will be made available upon reasonable request to the corresponding author. This retrospective study was approved by the institutional review board with a waiver of informed consent. This research follows principles outlined in the Declaration of Helsinki. The results of this study were reported in accordance with the Strengthening the Reporting of Observational Studies in Epidemiology (STROBE) guidelines. 

Cohort identification

A retrospective review was conducted of patients admitted to the trauma service at a level 1 regional trauma center (Cooper University Health Care, Camden, NJ, USA) between April 2020 and December 2022. Inclusion criteria were (i) admission diagnosis code of cerebral infarction (ICD-10 Code I63); (ii) CTA head and neck within less than 24 hours of hospital arrival; (iii) CTA contemporaneously analyzed with Viz LVO; (iv) no intracranial hemorrhage (ICH); (v) radiologist-confirmed intracranial occlusion. Only cases with CTA performed within 24 hours of hospitalization were included to exclude patients who developed in-hospital post-traumatic intracranial occlusion [21]. Patients presenting concomitantly with ICH were also excluded, as the acute management strategies for LVO and ICH are different [22]. 

Clinical protocol

For trauma patients, an institutional protocol for obtaining CTA head and neck upon arrival is based upon the risk factors described in the Modified Denver Criteria for blunt cerebrovascular injury (BCVI) [23]. Upon detection of potential intracranial occlusion by a radiologist, a multidisciplinary stroke team, consisting of neurologists, neurosurgeons, and/or neurointerventional providers, is activated for treatment planning, including administering thrombolysis agents and/or performing thrombectomy if appropriate. Interventional measures were pursued unless significant pre-hospital morbidity, contraindication, code status, or other advanced directives precluded further intervention. The thrombectomy technique, duration, devices used, and final recanalization status were determined at the discretion of the neurointerventional provider. 

With Viz LVO integration at our institution, the software automatically reviewed CTA head and neck images after the scan was completed. If Viz LVO identified an occlusion, it triggered a real-time alert to the multidisciplinary stroke team for prioritized evaluation [24].

Data collection

Contemporaneous clinical documentation, imaging reports, and records from the Viz LVO software were the primary sources for data collection. Demographic variables collected included age at the time of encounter; sex; type of trauma; occlusion location; occlusion laterality; and Alberta Stroke Program Early Computed Tomography Score (ASPECTS) for anterior circulation infarcts. The rate of Viz LVO identification, arrival-to-imaging, and arrival-to-stroke team activation times were collected. If thrombectomy was performed, door-to-puncture time; puncture-to-revascularization time; and rate of successful revascularization (modified thrombolysis in cerebral infarction [mTICI] 2B/2C/3) were collected. Clinical outcome data collected included: premorbid and 90-day modified Rankin scores (mRS); baseline, 24-hour post-interventional (if applicable), and discharge National Institutes of Health Stroke Scale scores (NIHSS); and 90-day mortality rate. 

Outcomes

Primary outcomes reported were the percent detection of LVO (i.e., intracranial internal carotid artery (ICA) and M1/M2 segments of middle cerebral artery (MCA)) [25] and arrival-to-stroke team activation times. Exploratory post hoc analyses include the rate of successful recanalization, if thrombectomy was attempted, and 90-day neurological functional outcomes. 

Statistical analysis

Patients were analyzed cumulatively and were sub-analyzed further by whether the patient had an LVO or non-LVO intracranial occlusion. Additional exploratory analyses for interventional and clinical outcome data were conducted with the cumulative patient population. Continuous variables were reported as means and standard deviations or medians and interquartile ranges (IQR) if the distribution was non-normal. Categorical variables were reported as frequencies. Comparative analyses or other pre-specified statistical tests for significant differences were not performed due to the descriptive nature of the study. Discharge NIHSS scores were imputed to the highest value (i.e., 42) if the patient had died prior to discharge; no other data imputation was performed for missing data. Analyses were conducted using JMP v17.0.0 (SAS Institute Inc., Carey, NC, USA).

## Results

Demographics 

Of 9893 patients admitted to the trauma department within the inclusion timeframe, 88 (0.89%) patients had a cerebral stroke diagnosis at any time during admission. Ten patients (10/88; 11.4%) met inclusion criteria. The median age was 70 (IQR 58-87), and most patients were admitted following a fall (8/10; 80%) (Table 1). Six (6/10; 60.0%) patients had LVOs, and most LVOs occurred within the MCA (5/6; 83.3%) (Table 1).

**Table 1:** Baseline demographic data LVO, large vessel occlusion; IQR, interquartile range; mRS, modified Rankin score; NIHSS, National Institutes of Health Stroke Scale; ASPECTS, Alberta Stroke Program Early Computed Tomography Score.

Variable	Overall, n=10	LVO, n=6	No-LVO, n=4
Demographics			
Age (years), median (IQR)	70 (58-87)	70 (58-81)	76 (53-89)
Sex, no. (%)			
Female	6 (60.0%)	4 (66.7%)	2 (50.0%)
Male	4 (40.0%)	2 (33.3%)	2 (50.0%)
Type of Trauma, no. (%)			
Fall	8 (80.0%)	5 (83.3%)	3 (75.0%)
Motor Vehicle Accident	2 (20.0%)	1 (16.7%)	1 (25.0%)
Premorbid mRS, median (IQR)	1 (0-3)	1 (0-3)	1 (0-3)
Admission NIHSS, median (IQR)	24 (20-32)	23 (20-25)	33 (14-33)
Stroke Characteristics			
Occlusion Location, no. (%)			
Intracranial Internal Carotid Artery	1 (10.0%)	1 (16.7%)	0 (0.0%)
M1/M2 Middle Cerebral Artery	5 (50.0%)	5 (83.3%)	0 (0.0%)
Posterior Cerebral Artery/Basilar Artery	4 (40.0%)	0 (0.0%)	4 (100%)
Side of Occlusion, no. (%)			
Right	0 (0.0%)	0 (0.0%)	0 (0.0%)
Left	6 (60.0%)	6 (100%)	0 (0.0%)
Posterior	4 (40.0%)	0 (0.0%)	4 (100%)
ASPECTS, median (IQR)	7 (5-8)	7 (5-8)	N/A

Automated LVO detection

**Figure 1:**
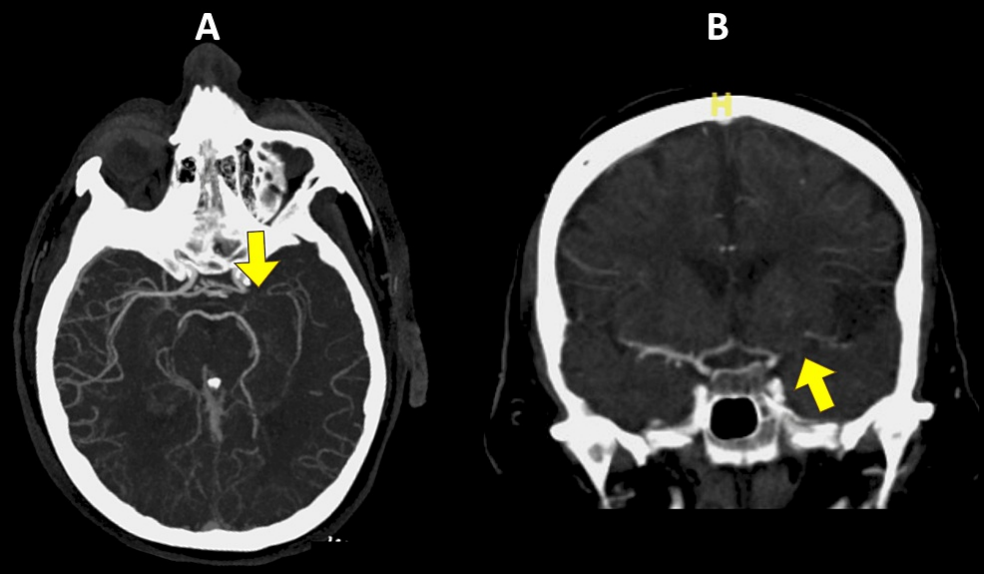
Successful identification by Viz LVO A 67-year-old female patient presented with left M1 MCA occlusion after a fall. The admission NIHSS score was 15. She was successfully revascularized with thrombectomy. The 24-hour post-intervention NIHSS score was 3. A) Viz LVO identification of M1 occlusion (arrow) on the axial view of CT angiography helical reconstruction. B) Coronal view of non-contrast CT angiography with M1 occlusion (arrow). LVO, large vessel occlusion; MCA, middle cerebral artery; M1, M1 segment of the middle cerebral artery; NIHSS, National Institute of Health Stroke Scale; CT, computed tomography.

**Figure 2:**
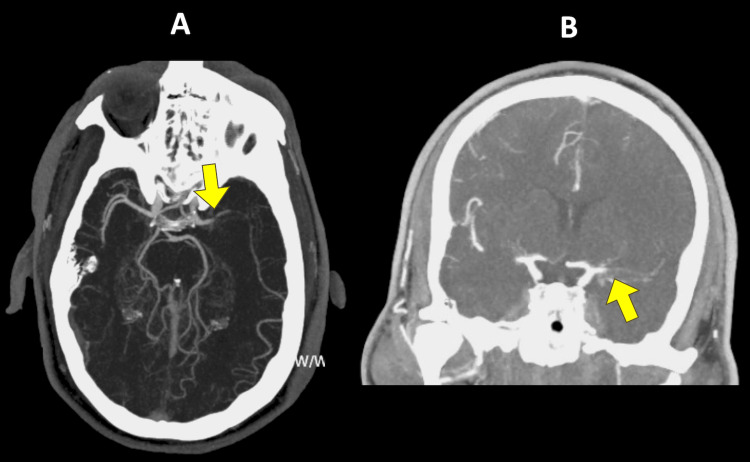
Missed identification by Viz LVO A 47-year-old male patient presented with a left M1 MCA occlusion after motor vehicle collision. The admission NIHSS score was 21. He was successfully revascularized with thrombectomy. The 24-hour post-intervention NIHSS score was 12. A) Viz LVO identification of M1 occlusion (arrow) on axial view of CT angiography helical reconstruction. B) Coronal view of non-contrast CT angiography with M1 occlusion (arrow). LVO, large vessel occlusion; MCA, middle cerebral artery; M1, M1 segment of the middle cerebral artery; NIHSS, National Institutes of Health Stroke Scale; CT, computed tomography.

**Figure 3:**
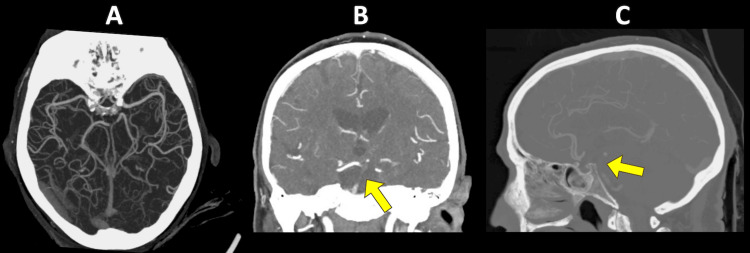
Occlusion not correctly identified as a large vessel occlusion by Viz LVO A 86-year-old female patient presented with basilar occlusion after a fall. Admission NIHSS score was 8. She was successfully revascularized with thrombectomy. The 24-hour post-intervention NIHSS score was 1. A) Viz LVO analysis on axial view of CT angiography helical reconstruction. B) Coronal view of non-contrast CT angiography with basilar occlusion (arrow). C) Sagittal view of non-contrast CT angiography with basilar occlusion (arrow). LVO, large vessel occlusion; NIHSS, National Institute of Health Stroke Scale; CT, computed tomography.

In patients with radiologist-confirmed LVOs, Viz LVO was able to correctly detect 83.3% (5/6) of patients (Table 2) (Figures 1A, 1B). The one LVO patient not detected by Viz LVO had an M1 MCA occlusion (Figures 2A, 2B). The stroke team was activated after a median of 41 minutes (IQR 13-120) following the arrival for LVO stroke patients (Table 2). Among patients with non-LVO intracranial occlusions, Viz LVO did not falsely identify the occlusion as an LVO for any patients (Table 2) (Figures 3A-3C). The stroke team was activated after a median of 98 minutes (IQR 29-262) following the arrival of a non-LVO stroke patient (Table 2).

**Table 2:** Automated stroke detection and response-activation data LVO, large vessel occlusion; IQR, interquartile range; min, minutes.

Variable	Overall, n=10	LVO, n=6	No-LVO, n=4
Viz LVO Detected, no. (%)	5 (50.0%)	5 (83.3%)	0 (0.0%)
Arrival-to-Imaging Time (min), median (IQR)	26 (21-39)	27 (24-44)	20 (12-48)
Arrival-to-Stroke Team Time (min), median (IQR)	47 (21-133)	41 (13-120)	98 (29-262)
Thrombectomy, no. (%)	7 (70.0%)	4 (66.7%)	3 (75.0%)

Exploratory post hoc analyses

Mechanical Thrombectomy Outcomes

Seven (7/10; 70.0%) patients were treated with mechanical thrombectomy (Table 2). Median arrival-to-puncture time was 146 minutes (IQR 87-310) (Table 3). Successful revascularization was achieved in 85.7% (6/7) patients, and the average improvement in the NIHSS score from admission to 24 hours post-intervention was 6 (IQR 0-9) (Table 3).

**Table 3:** Procedural data for patients treated with mechanical thrombectomy min, minutes; IQR, interquartile range; mTICI, modified thrombolysis in cerebral infarction score; hr, hour; NIHSS, National Institutes of Health Stroke Scale.

Variable	n=7
Arrival-to-Puncture Time (min), median (IQR)	146 (87-310)
Puncture-to-Revascularization Time (min), median (IQR)	24 (11-36)
Successful Revascularization (mTICI 2B/2C/3), no. (%)	6 (85.7%)
24hr NIHSS Score, median (IQR)	16 (3-33)
NIHSS Change (Admission Score - 24hr Score), median (IQR)	6 (0-9)

Clinical Outcomes and Mortality

Seven (7/10; 70.0%) patients ultimately died before discharge (Table 4). After 90 days, only two (2/10; 20.0%) patients survived, and both patients had achieved good neurological recovery (mRS 0-2) (Table 4). These two patients had been successfully treated with mechanical thrombectomy. 

**Table 4:** Clinical outcome data NIHSS, National Institute of Health Stroke Scale; mRS, modified Rankin score.

Variable	n=10
Discharge NIHSS, median (IQR)	42 (5-42)
90 day mRS 0-2, no. (%)	2 (20.0%)
In-Hospital Mortality, no. (%)	7 (70.0%)
Survival at 90 days, no. (%)	2 (20.0%)

## Discussion

Our analysis describes an early experience with Viz LVO for identifying acute ischemic stroke upon admission in trauma patients at a level one trauma center. The Viz LVO software was able to correctly identify 83.3% (5/6) LVOs in our cohort, and these patients were triaged by the stroke team within an average time of less than one hour from arrival. A prior, single-center retrospective study of trauma patients from 2018 demonstrated that 5.7% of patients were diagnosed with ischemic stroke [4,5]. However, no further stroke workup was pursued on admission for any of the patients, despite a few patients presenting with neurological deficits, and the median time to neurological evaluation and diagnosis was approximately two days [4,5]. Our contrasting experience provides preliminary data supporting the potential usefulness of applying automated stroke detection tools to the trauma subpopulation. 

Thrombectomy data was explored in our trauma cohort. Treatment for LVOs is time-sensitive, with a goal thrombectomy time from arrival to puncture to be below 90 minutes [26]. The average arrival-to-puncture time in our cohort did not achieve this goal; further analyses in larger trauma cohorts will elucidate reasons for delay and opportunities to optimize clinical workflow. Although the effectiveness of thrombectomy is not as well-characterized in the trauma population, prior limited studies have described the feasibility of this intervention, and the association between timely treatment with thrombectomy and favorable neurological recovery for patients with severe traumatic injury [5,13,14]. Triage within the thrombectomy eligibility window is challenging in the absence of a neurological exam, particularly if the patient is obtunded or sedated [4,5]. Consequently, our experience supports the concept that automated image analysis tools may have a role in aiding timely stroke identification, particularly when clinical evaluation is limited, for facilitating early treatment in this diagnostically challenging subpopulation. 

A few patients in our trauma cohort had intracranial occlusions in the basilar artery or posterior cerebral artery. However, Viz LVO and other similar commercially available, automated stroke identification tools are typically not designed to identify occlusions within these segments [15,19,24]. With recent data supporting the benefits of thrombectomy in basilar stroke patients [27], further augmenting existing automated detection tools to include non-LVO occlusions would be particularly beneficial in trauma patients to aid timely identification of both LVO and non-LVO thrombectomy candidates.

Limitations

Given that this was a retrospective analysis at a single institution, this analysis is subject to limitations inherent to the study design. Given the small sample size, the analysis was primarily descriptive in nature. Future investigations with a larger study cohort would further help characterize the accuracy and clinical impact of automated stroke detection analysis tools within the trauma subpopulation. Furthermore, the high mortality rate observed in our cohort likely reflects the overall difficulty of stroke rehabilitation in a population with concomitant traumatic injuries. With a larger sample size, matching study cohorts by traumatic injury severity would more aptly control for the effect of injury severity on long-term clinical outcomes. Additionally, our analysis was restricted to stroke identification during admission, and future studies may expand upon our analysis to assess the benefit of automated stroke detection tools for patients with new ischemic stroke occurring in the post-traumatic period. 

## Conclusions

Detecting ischemic stroke in the trauma subpopulation upon admission is challenging due to several concomitant factors. Consequently, identifying adjunct tools to aid identification and timely treatment response to ischemic stroke is necessary to increase the chance for meaningful neurological recovery. At a level one trauma center, our early experience with Viz LVO, one of the commercially available software programs, automatically processing CTA images showed its potential for aiding occlusion identification and subsequent stroke team mobilization. Future studies in larger cohorts will expand upon these preliminary findings to establish the accuracy and clinical benefit of automated stroke detection tool integration for the trauma population. 
